# Actinomycetes from the South China Sea sponges: isolation, diversity, and potential for aromatic polyketides discovery

**DOI:** 10.3389/fmicb.2015.01048

**Published:** 2015-10-01

**Authors:** Wei Sun, Fengli Zhang, Liming He, Loganathan Karthik, Zhiyong Li

**Affiliations:** Marine Biotechnology Laboratory, State Key Laboratory of Microbial Metabolism, School of Life Sciences and Biotechnology, Shanghai Jiao Tong UniversityShanghai, China

**Keywords:** marine sponges, actinomycetes, diversity, aromatic polyketides, KS_α_ gene

## Abstract

Marine sponges often harbor dense and diverse microbial communities including actinobacteria. To date no comprehensive investigation has been performed on the culturable diversity of the actinomycetes associated with South China Sea sponges. Structurally novel aromatic polyketides were recently discovered from marine sponge-derived *Streptomyces* and *Saccharopolyspora* strains, suggesting that sponge-associated actinomycetes can serve as a new source of aromatic polyketides. In this study, a total of 77 actinomycete strains were isolated from 15 South China Sea sponge species. Phylogenetic characterization of the isolates based on 16S rRNA gene sequencing supported their assignment to 12 families and 20 genera, among which three rare genera (*Marihabitans, Polymorphospora*, and *Streptomonospora*) were isolated from marine sponges for the first time. Subsequently, β-ketoacyl synthase (KS_α_) gene was used as marker for evaluating the potential of the actinomycete strains to produce aromatic polyketides. As a result, KS_α_ gene was detected in 35 isolates related to seven genera (*Kocuria, Micromonospora, Nocardia, Nocardiopsis, Saccharopolyspora, Salinispora*, and *Streptomyces*). Finally, 10 strains were selected for small-scale fermentation, and one angucycline compound was detected from the culture extract of *Streptomyces anulatus* strain S71. This study advanced our knowledge of the sponge-associated actinomycetes regarding their diversity and potential in producing aromatic polyketides.

## Introduction

As one of the oldest multicellular animals (Love et al., 2009), marine sponges (phylum Porifera) often harbor dense and diverse microbial communities, and the sponge-microbe associations represent one of the most complex symbioses on earth (Taylor et al., 2007). Actinobacteria are commonly found in association with sponges (Simister et al., 2012). In the past decade, extensive efforts have been made in isolating actinomycetes from sponges (Zhang et al., 2006; Abdelmohsen et al., 2010, 2014b; Vicente et al., 2013). To date, at least 60 actinobacterial genera have been set apart from marine sponges (Abdelmohsen et al., 2014a). The investigations on the culturable diversity of sponge-associated actinomycetes not only advanced our knowledge of those actinomycetes in special habitats but also provided new opportunities for natural product search and discovery (Abdelmohsen et al., 2014a). In China oceans, the largest group of sponges inhabits the South China Sea (Zhang et al., 2003). To our knowledge, in previous studies 15 actinobacterial genera have been isolated from South China Sea sponges (Jiang et al., 2007, 2008; Sun et al., 2010; Li et al., 2011; Xi et al., 2012). Nevertheless, previous cultivation attempts were set to a few South China Sea sponge species out of thousands of South China Sea sponges, which probably underestimated the culturable diversity of sponge-associated actinomycetes. Thus, collecting as many sponges as possible from the South China Sea is significant to comprehensively explore their associated actinomycetes.

Previous surveys have demonstrated that sponges are chemically defended from predation and marine pathogens either by the compounds they produce or those produced by symbionts or associated microorganisms (Puglisi et al., 2014). Actinomycetes are known to produce aromatic polyketides by type II polyketide pathway (Schneider, 2005). Actinomycete-derived aromatic polyketide compounds have exhibited a wide range of bioactivities and clinical importance (Hertweck et al., 2007). Notably, a few anthracyclines and tetracyclines have emerged as clinical drugs for decades, such as doxorubicin (antineoplastic) and tetracycline (antibiotic). Furthermore, many of these compounds are promising drug candidates (Hertweck et al., 2007). Therefore, sponge-associated actinomycetes may provide chemical defense for their hosts by producing aromatic polyketides. Recently, in exploring new sources of aromatic polyketides the sponge-associated actinomycetes warranted particular attention. Particularly, a few structurally novel aromatic polyketides were discovered from sponge-associated actinomycetes such as *Saccharopolyspora* and *Streptomyces* strains (Perez et al., 2009; Motohashi et al., 2010; Schneemann et al., 2010a). In view of the remarkable diversity of sponge-associated actinomycetes, the producers of aromatic polyketides are not merely limited to *Saccharopolyspora* and *Streptomyces*. Thus, we opine that the potential of sponge-associated actinomycetes in producing aromatic polyketides is underexplored and it is worth investigating in depth.

Over the past decade, sequence-guided genetic screening strategy has been used in the discovery of certain compound classes from actinomycetes, such as halometabolites (Hornung et al., 2007), type I polyketides (Gontang et al., 2010), and phenazines (Karuppiah et al., 2015), indicating that a small amount of sequence from appropriate genetic loci can be used to predict secondary-metabolite production in cases where the sequences have high identity level to experimentally characterized biosynthetic pathways. The gene-compound route has become a feasible approach for natural product search and discovery. Therefore, genetic screening strategy together with small-scale fermentation and chemical analyses was used in this study to specifically search for aromatic polyketides.

In this work, we aimed to investigate the culturable diversity of sponge-associated actinomycetes from the South China Sea and explore the potential use of the sponge-associated actinomycetes as a novel source of aromatic polyketides. As a result, we cultivated as many as 20 actinomycete genera, screened seven genera as potential producers of aromatic polyketides and identified one angucycline compound from a *Streptomyces* strain. This study advanced our knowledge of South China Sea sponge-associated actinomycetes in respect to their diversity and metabolic potential of aromatic polyketides.

## Materials and methods

### Sample collection

A total of 15 sponge species were collected by scuba diving from the South China Sea, including six at a depth of 5–10 m from coastal waters, respectively Sanya Bay (18°13′N; 109°29′E), Xinying Harbor (19°90′N; 109°52′E), and Xincun Harbor (18°40′N; 110°00′E) and nine at a depth of 10–20 m from a remote island, Yongxing Island (16°50′N; 112°20′E) (Table 1). The sponges were identified based on their morphology or 18S rRNA gene/internal transcribed spacer (ITS) sequences (Table 1). The samples were placed into plastic bags and transported to the laboratory using ice box, then stored at *z*−20°C until analysis.

**Table 1:** **Sponge samples collected from the South China Sea and their actinomycete isolates**.

**Sponge species**	**Identification method (NCBI accession no.)**	**Geographical location**	**Collection month**	**No. of isolates**	**No. of genera**
*Haliclona* sp.	morphology	Sanya Bay	2009.07	11	6
*Trachycladus laevispirulifer*	morphology	Xinying Harbor	2010.06	3	3
*Amphimedon queenslandica*	ITS sequence (KC762728)	Xincun Harbor	2011.05	6	3
*Haliclona mediterranea*	18S rRNA gene sequence (KC762723)	Xincun Harbor	2011.05	3	2
*Lamellodysidea* sp.	ITS sequence (KC762730)	Xincun Harbor	2011.05	8	5
*Cliona* sp.	ITS sequence (KC762729)	Xincun Harbor	2011.05	5	2
*Phyllospongia foliascens*	morphology	Yongxing Island	2011.05	1	1
*Agelas clathrodes*	18S rRNA gene sequence (KC762715)	Yongxing Island	2011.05	2	2
*Ircinia felix*	18S rRNA gene sequence (KC762716)	Yongxing Island	2011.05	2	2
*Hippospongia lachne*	18S rRNA gene sequence (KC762719)	Yongxing Island	2011.05	2	1
*Cinachyrella* sp.	18S rRNA gene sequence (KC762720)	Yongxing Island	2011.05	5	4
*Aplysina fistularis*	18S rRNA gene sequence (KC762723)	Yongxing Island	2011.05	8	2
*Arenosclera heroni*	18S rRNA gene sequence (KJ675584)	Yongxing Island	2013.07	5	4
*Plakortis simplex*	morphology	Yongxing Island	2013.07	12	4
*Phakellia fusca*	morphology	Yongxing Island	2013.07	4	3

### Isolation of actinomycetes

Five media were used for the isolation of sponge-associated actinomycetes (Table S1), four of which were chosen based on previous studies on the culturable diversity of marine sediment-derived and sponge-associated actinomycetes (Mincer et al., 2002; Zhang et al., 2006; Abdelmohsen et al., 2010) and one was designed in this study. All media were supplemented with K_2_Cr_2_O_7_ (50 μgml^−1^) to inhibit fungi and nalidixic acid (15 μgml^−1^) to inhibit Gram-negative bacteria. Sponge samples were rinsed with sterile artificial seawater (26.52 g NaCl, 5.228 g MgCl_2_6H_2_O, 3.305 g MgSO_4_, 1.141 g CaCl_2_, 0.725 g KCl, 0.202 g NaHCO_3_, 0.083 g NaBr, 1 L distilled water) to remove the microbes loosely attached on the surface. Subsequently, a few tissue cubes were excised from different sections (including cortex and endosome) of the sponge samples. They were cut into pieces and aseptically ground using sterilized pestles and mortars. Actinomycetes were isolated by means of serial dilution and plating techniques. The inoculated plates were incubated at 28°C for 3–6 weeks. The colonies bearing distinct morphological characteristics were picked up and transferred onto freshly prepared media until pure cultures were obtained.

### Genomic DNA extraction

To prepare cultures for the extraction of genomic DNA from the isolates, a single colony was transferred to a 5 ml microtube with 1 ml of liquid medium from which the isolate was originally picked up. The cultures were incubated for 3–5 days at 28°C with shaking at 180 rpm. Bacterial cells from these cultures were collected by centrifugation and genomic DNA was extracted as described by Sun et al. (2010).

### PCR amplification and sequencing of 16S rDNA

The *Actinobacteria*-specific primers S-C-Act-0235-a-S-20 (5′-CGCGGCCTATCA GCTTGTTG-3′) and S-C-Act-0878-a-A-19 (5′-CCG TACTCCCCAGGCGGGG-3′) were used for the amplification of actinobacterial 16S rRNA gene fragment (Stach et al., 2003). Cycling conditions were as follows: initial denaturation at 95°C for 4 min, 30 cycles of 95°C for 45 s, 68°C for 45 s, and 72°C for 1 min, and a final extension of 5 min at 72°C. Subsequently, the universal bacterial primers 27F (5′-GAGTTTGATCCTG GCTCAG-3′) and 1500R (5′-AGAAAGGAG GTGATCCAGCC-3′) were used to amplify nearly complete 16S rRNA gene of the actinomycete candidates (Woese et al., 1983). Cycling conditions were as follows: initial denaturation at 95°C for 3 min, 30 cycles of 94°C for 30 s, 54°C for 40 s, and 72°C for 2 min, and a final extension of 10 min at 72°C. The PCR products were purified and sequenced on the ABI 3730 automated sequencer at Majorbio Bio-Pharm Technology Co. Ltd. (Shanghai).

### PCR amplification, cloning, and sequencing of KS gene

To screen aromatic polyketide producers from all the isolates, the degenerate primers IIPF6 (5′-TSGCSTGCTTCG AYGCSATC-3′) and IIPR6 (5′-TGGAANCCGCCGAAB CCGCT-3′) were used to amplify type II polyketide KS_α_ gene fragment (Metsä-Ketelä et al., 1999). This primer pair was reported to be favorable for the majority of known KS_α_ gene and previously used in the investigation on marine sponge-associated actinobacteria (Schneemann et al., 2010b). Cycling conditions were as follows: initial denaturation at 95°C for 5 min, 30 cycles of 95°C for 35 s, 55°C for 40 s, and 72°C for 1 min, and a final extension of 10 min at 72°C. The amplified products of approximately 600 bp were recovered and purified using Agarose Gel DNA Purification Kit (Takara, Dalian). Purified PCR products were cloned into pMD18-T vector (Takara, Dalian) and transformed into CaCl_2_-competent *Escherichia coli* DH5α. The positive recombinants were screened on X-Gal-IPTG-ampicillin plates. Respectively five positive clones were randomly selected from each library and sequenced using M13F primer on the ABI 3730 automated sequencer at Sangon Biotech Co. Ltd. (Shanghai).

### Sequence analysis

All the sequence data were proofread using Chromas, version 1.62 (Technelysium). The 16S rRNA gene sequences were compared with those from the type strains available in NCBI (http://www.ncbi.nlm.nih.gov/) using the Basic Local Alignment Search Tool (BLAST) (Altschul et al., 1990). For KS_α_ gene analysis, the nucleotide sequences were translated to amino acid sequences using the web tool ORF Finder in NCBI (http://www.ncbi.nlm.nih.gov/projects/gorf/). The deduced amino acid sequences were compared with the KS_α_ sequences in PKMiner database (http://www.webcitation.org/6C9a5WoFY) using the type II PKS domain classifiers (Kim and Yi, 2012). The top matches were derived from the KS_α_ sequences associated with 42 experimentally characterized pathways. For phylogenetic analysis, multiple sequence alignment was performed using CLUSTALX, version 1.81. Phylogenetic tree was constructed using Mega 4.1 (Tamura et al., 2007). The consistency of the trees was verified by bootstrapping (1000 replicates) for parsimony.

### Small-scale fermentation

To test the production of aromatic polyketides, small-scale fermentation studies were performed targeting 10 representative strains, which were selected based on KS_α_ sequence analyses. They were grown in 250 ml Erlenmeyer flasks each containing 100 ml of medium GYM4 (10 g glucose, 4 g yeast extract, 4 g malt extract, 1 liter water, pH 7.2) for 5 days at 28°C with shaking (at 120 rpm) in the dark. Each culture was inoculated separately with a 1 cm^2^ piece from a culture grown on a GYM4 agar plate for 2 weeks at 28°C in the dark.

### Chemical analysis of culture extracts

After mycelium was removed by vacuum filtration, the fermentation broth was extracted with 100 ml of acetic ether (EtOAc) and taken to dryness by rotary evaporation. EtOAc extract was dissolved in methanol for HPLC-DAD analysis on an Agilent 1200 series (Agilent Technologies, USA) with an Diode Array Detector (DAD) and a C18 RP-column (Eclipse XDB-C18 5 μm, 4.6 × 150 mm), with a gradient from 5% acetonitrile in water to 100% acetonitrile over 20 min. Ultraviolet-visible (UV-vis) absorption spectra ranging from 200 to 600 nm of the components in each crude extract were examined. Compounds owning characteristic UV-vis absorption of aromatic polyketides were searched and designated as putative candidates. Prior to LC/MS analysis, the compound candidates were preliminarily separated from the crude extracts by semi-preparative HPLC with methanol gradient elution. This procedure was conducted on an Agilent 1200 series (Agilent Technologies, USA) with a variable wavelength detector (VWD) and a C18 RP-column (Unitary C18 5 μm, 10 × 250 mm).

Collected fractions were dried in vacuo and dissolved in methanol for LC/MS analysis. The fractions were detected on an ultra-performance liquid chromatography and quadrupole time of flight mass spectroscopy (UPLC-QTOF-MS Premier, Waters Corporation, USA). The analytes were separated on a C18 RP-column (ACQUITY BEH-C18 1.7 μm, 2.1 × 100 mm, Waters Co.) with methanol gradient elution. High-resolution mass spectrum (HR-MS) of target ion was acquired in positive electro-spray ionization mass spectrum (ESI-MS) mode.

MS data was analyzed using the software MassLynx. The major ion peaks with a mass range of 300–1000 Da were preferentially selected. Corresponding to each peak ([M+H]^+^ or [M+Na]^+^), a few suggested molecular formula were obtained. After those not matching aromatic polyketide compounds were excluded, the remaining ones were used as queries (subtracting one H or Na) to match reported aromatic polyketides in SciFinder database (https://scifinder.cas.org/scifinder/). For those retrieved compounds, their UV-vis absorption spectra were compared with our target substance.

### Nucleotide sequence accession numbers

The sequences obtained in this study were deposited to GenBank with the 16S rRNA gene sequences under the accession numbers: JX007945–JX008000, KJ094386–KJ094406 and the KS_α_ gene sequences under the numbers: JX008002–JX008015, KJ094407–KJ094410.

## Results

### Culture-dependent diversity of sponge-associated actinomycetes

In this study, a total of 77 isolates were identified as actinomycetes, which were assigned to 12 families and 20 genera (Table 2). Among the 20 genera, *Micromonospora, Mycobacterium, Nocardia, Nocardiopsis, Pseudonocardia, Rhodococcus, Salinispora*, and *Streptomyces* were previously isolated from South China Sea sponges (Jiang et al., 2007, 2008; Sun et al., 2010; Li et al., 2011; Xi et al., 2012), the other 12 genera marked in Table 2 were cultivated from South China Sea sponges first time. Based on the latest reviews (Abdelmohsen et al., 2014a; Valliappan et al., 2014) and our retrievals of sponge-derived 16S rRNA gene sequences in GenBank, we found this was the first report of three rare genera, i.e., *Marihabitans, Polymorphospora*, and *Streptomonospora*, isolated from marine sponges.

**Table 2:** **Molecular identification of the actinomycetes from South China Sea sponges based on 16S rRNA gene and KS_α_ gene detection**.

**Family**	**Genus**	**OUT no**.	**Strain (NCBI accession no.)**	**Nearest type strain (NCBI accession no.)**	**Identity (%)**	**PKS II**
*Brevibacteriaceae*	Brevibacterium^*^	1	S49 (JX007974)	*B. linens* (NR_026166)	99.3	−
*Dermabacteraceae*	Brachybacterium^*^	2	S26 (JX007960)	*B. squillarum* (GQ339911)	99.5	−
*Intrasporangiaceae*	Marihabitans^*^	3	S53 (JX007977)	*M. asiaticum* (NR_041559)	100	−
	Serinicoccus^*^	4	S11 (JX007953)	*S. chungangensis* (HM068886)	98.7	−
		4	S24 (JX007958)	*S. chungangensis* (HM068886)	98.7	−
		4	S38 (JX007966)	*S. chungangensis* (HM068886)	99.0	−
		4	S69 (JX007986)	*S. chungangensis* (HM068886)	98.6	−
*Microbacteriaceae*	Microbacterium^*^	5	S15 (JX007956)	*M. chocolatum* (AM181503)	99.8	−
		5	S25 (JX007959)	*M. chocolatum* (AM181503)	99.9	−
*Micrococcaceae*	Arthrobacter^*^	6	S70 (JX007987)	*A. protophormiae* (NR_026195)	99.8	−
	Kocuria^*^	7	S12^#^ (JX007954)	*K. gwangalliensis* (EU286964)	96.8	+
		8	S14 (JX007955)	*K. turfanensis* (NR_043899)	98.8	−
		8	S42 (JX007970)	*K. turfanensis* (NR_043899)	98.6	−
		8	S50 (JX007975)	*K. turfanensis* (NR_043899)	98.7	−
		8	S61 (JX007984)	*K. turfanensis* (NR_043899)	98.7	−
		9	S43 (JX007971)	*K. flava* (NR_044308)	99.7	−
		10	S45 (JX007972)	*K. palustris* (NR_026451)	100	−
		10	S62 (JX007985)	*K. palustris* (NR_026451)	99.9	−
		11	S48 (JX007973)	*K. marina* (NR_025723)	99.7	−
	Micrococcus^*^	12	S23 (JX007957)	*M. endophyticus* (NR_044365)	99.8	−
*Micromonosporaceae*	*Micromonospora*	13	S60 (JX007983)	*M. aurantiaca* (NR_074415)	99.6	−
		13	S80 (JX007997)	*M. aurantiaca* (NR_074415)	99.5	−
		13	S97^#^ (KJ094396)	*M. aurantiaca* (NR_074415)	99.4	+
	Polymorphospora^*^	14	S07 (JX007949)	*Polymorphospora* sp. (NR_044592)	98.7	−
		14	S09 (JX007951)	*Polymorphospora* sp. (NR_044592)	98.8	−
		15	S85 (KJ094387)	*P. rubra*(NR_041314)	100	−
	*Salinispora*	16	S06 (JX007948)	*S. arenicola* (NR_074612)	99.9	+
		16	S08 (JX007950)	*S. arenicola* (NR_074612)	100	+
		16	S32 (JX007962)	*S. arenicola* (NR_074612)	99.8	+
		16	S33^#^ (JX007963)	*S. arenicola* (NR_074612)	99.7	+
		16	S55 (JX007979)	*S. arenicola* (NR_074612)	99.9	+
		16	S56 (JX007980)	*S. arenicola* (NR_074612)	99.9	+
		16	S58 (JX007981)	*S. arenicola* (NR_074612)	99.9	+
		16	S83 (JX008000)	*S. arenicola* (NR_074612)	100	+
		16	S84 (KJ094386)	*S. arenicola* (NR_074612)	100	+
		16	S87 (KJ094389)	*S. arenicola* (NR_074612)	99.9	+
		16	S93 (KJ094392)	*S. arenicola* (NR_074612)	99.8	+
		16	S94 (KJ094393)	*S. arenicola* (NR_074612)	100	+
		16	S99 (KJ094398)	*S. arenicola* (NR_074612)	99.9	+
		16	S100 (KJ094399)	*S. arenicola* (NR_074612)	100	+
		16	S102 (KJ094401)	*S. arenicola* (NR_074612)	99.9	+
		16	S108 (KJ094405)	*S. arenicola* (NR_074612)	100	+
		17	S34^#^ (JX007964)	*S. tropica* (NR_074502)	99.5	+
		17	S54 (JX007978)	*S. tropica* (NR_074502)	99.5	−
		17	S96 (KJ094395)	*S. tropica* (NR_074502)	99.5	−
		17	S98 (KJ094397)	*S. tropica* (NR_074502)	99.4	−
		17	S101 (KJ094400)	*S. tropica* (NR_074502)	99.5	−
		17	S103 (KJ094402)	*S. tropica* (NR_074502)	99.4	−
*Mycobacteriaceae*	*Mycobacterium*	18	S01 (JX007945)	*M. poriferae* (NR_025235)	98.9	−
		18	S02 (JX007946)	*M. poriferae* (NR_025235)	98.9	−
*Nocardiaceae*	*Nocardia*	19	S107^#^ (KJ094404)	*N. araoensis* (NR_028652)	98.6	+
	*Rhodococcus*	20	S106 (KJ094403)	*R. opacus*(NR_074632)	98.2	−
		20	S109 (KJ094406)	*R. opacus*(NR_074632)	98.0	−
*Nocardiopsaceae*	*Nocardiopsis*	21	S77 (JX007994)	*N. alba* (NR_026340)	100	+
		21	S78^#^ (JX007995)	*N. alba* (NR_026340)	99.9	+
		22	S92^#^ (KJ094391)	*N. halotolerans* (NR_025422)	99.1	+
	Streptomonospora^*^	23	S05 (JX007947)	*S. halophila* (NR_044207)	97.7	−
*Pseudonocardiaceae*	*Pseudonocardia*	24	S76 (JX007993)	*P. carboxydivorans* (NR_044092)	99.2	−
	Saccharopolyspora^*^	25	S36^#^ (JX007965)	*S. gloriosae* (EU005371)	99.2	+
		25	S79 (JX007996)	*S. gloriosae* (EU005371)	99.4	+
*Streptomycetaceae*	*Streptomyces*	26	S10^#^ (JX007952)	*S. parvulus* (NR_041119)	99.8	+
		27	S31^#^ (JX007961)	*S. carnosus* (AB184263)	100	+
		28	S39^#^ (JX007967)	*S. djakartensis* (NR_041178)	99.5	+
		29	S40^#^ (JX007968)	*S. luteosporeus* (AB184607)	97.6	+
		30	S41^#^ (JX007969)	*S. rochei* (NR_041091)	100	+
		31	S52 (JX007976)	*S. flavofuscus* (DQ026648)	98.2	−
		32	S59 (JX007982)	*S. resistomycificus* (NR_042100)	99.8	−
		33	S71^#^ (JX007988)	*S. anulatus* (NR_041062)	99.8	+
		34	S72^#^ (JX007989)	*S. xiamenensis* (NR_044035)	99.4	+
		35	S73 (JX007990)	*S. sclerotialus* (NR_025620)	98.3	−
		35	S74 (JX007991)	*S. sclerotialus* (NR_025620)	98.2	−
		36	S75 (JX007992)	*S. albidoflavus* (NR_041095)	99.3	−
		37	S81^#^ (JX007998)	*S. diastaticus* (NR_043486)	99.6	+
		38	S82 (JX007999)	*S. marinus* (AB473554)	97.8	−
		39	S86^#^ (KJ094388)	*S. griseorubens* (NR_041066)	100	+
		39	S95 (KJ094394)	*S. griseorubens* (NR_041066)	100	+
*Streptosporangiaceae*	Nonomuraea^*^	40	S88 (KJ094390)	*N. ferruginea* (NR_025996)	98.7	−

The 12 genera marked with

* were cultivated from South China Sea sponges for the first time and the 17 strains marked with

# *were selected for KS_α_ gene analysis*.

**Figure 1:**
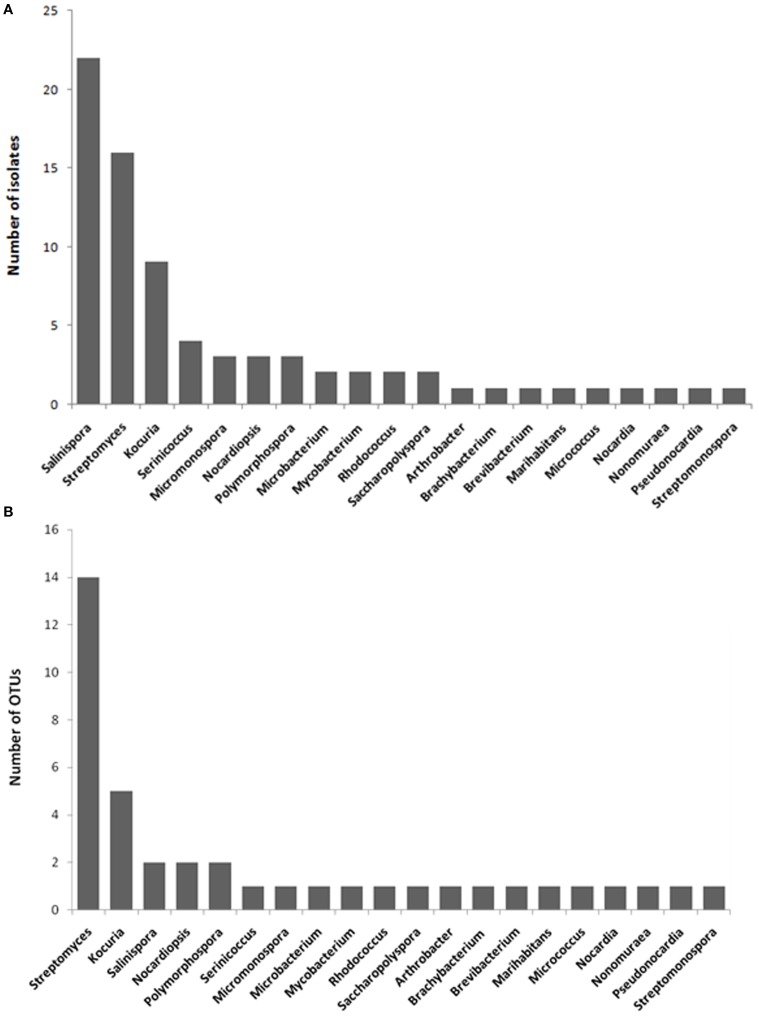
**Number of isolates (A) and OTUs per actinobacterial genus (B)**.

The highest number of the isolates was affiliated with *Salinispora*, followed by *Streptomyces, Kocuria, Serinicoccus, Micromonospora, Nocardiopsis, Polymorphospora*, and other genera (Figure 1A). The number of the isolates differed considerably among different marine sponges. *Plakortis simplex* yielded the highest number of isolates, followed by *Haliclona* sp., *Lamellodysidea* sp., *Aplysina fistularis, Amphimedon queenslandica*, and other sponges (Table 1). Similarly, the actinobacterial diversity at the genus level also varied as sponge species. The highest diversity was observed in *Haliclona* sp. with six genera cultivated, followed by *Lamellodysidea* sp. and other sponges (Table 1). The 77 isolates were assigned to 40 operational taxonomic units (OTUs) based on 99.5% sequence identity, representing 40 species. The most diverse group was *Streptomyces* with 14 OTUs obtained, followed by *Kocuria* and other genera (Figure 1B).

On the whole, *Streptomyces* and *Salinispora* were most common groups in the South China Sea sponges. The former was isolated from nine sponges and the latter from six sponges. *Streptomyces* was widespread in the sponges from distinct geographical locations whereas *Salinispora* was mainly distributed in the open sea sponges. Additionally, *Kocuria* was derived from four sponges inhabiting the same site, Xincun Harbor, indicating its distribution specificity.

### Structure diversity evaluation of putative aromatic polyketide products

**Figure 2:**
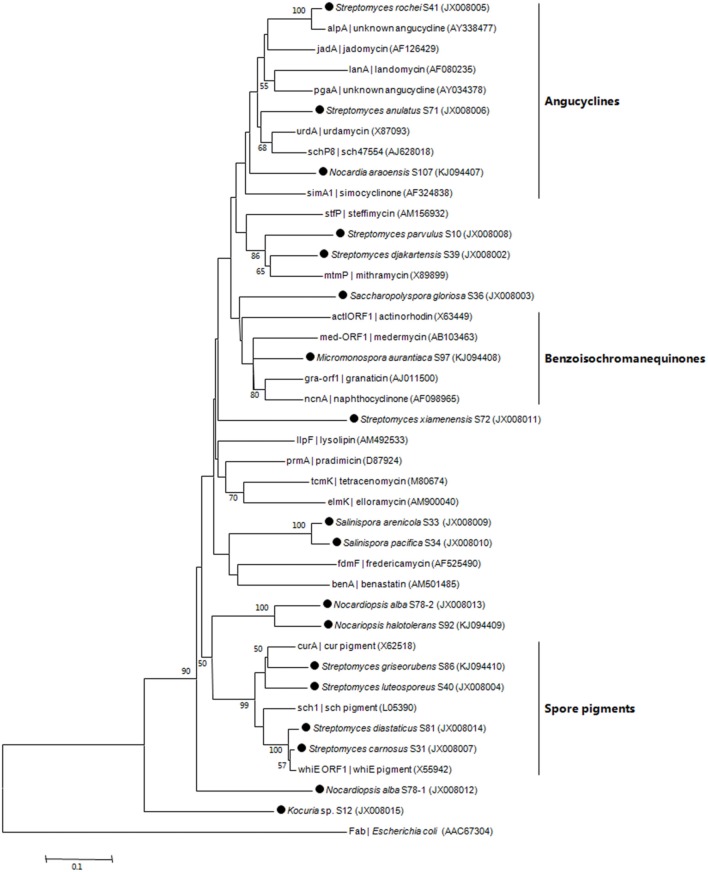
**Neighbor-joining tree constructed using aligned KS_α_ domain amino acid sequences (203 amino acid positions)**. The sequences obtained in this work are marked by black dot. Next to the KS_α_ gene name, the identified, or predicted compounds and GenBank accession number of the gene cluster are indicated. Boot strap values calculated from 1000 resamplings using neighborjoining are shown at the respective nodes when the calculated values were 50% or greater. The scale bar represents 0.1 substitutions per amino acid position.

PCR fragments of KS_α_ gene were amplified from 35 out of 77 isolates (Table 2). The 35 isolates were assigned to 17 OTUs. In total, 17 PCR fragments from 17 OTUs were selected for KS_α_ gene cloning and sequencing, and 18 unique sequences were obtained. Based on homology comparison (Table 3) and phylogenetic analysis (Figure 2), high structural diversity of putative aromatic polyketide products was observed, concerning different subtypes. Homology-based searches on the amino acid level indicated that the putative KS_α_ sequences, respectively displayed 85.2–100% maximum similarity to those KSs associated with experimentally characterized biosynthetic pathways (Table 3). By comparing those known KS_α_ sequences in PKMiner database, it was observed that most sequences grouped in the same subtype share ≥93.6% amino acid similarity with each other. Thus, this similarity was used as sequence clustering criterion in this work. Of the obtained 18 KS_α_ sequences, eight shared ≥93.6% similarity with their top matches, which were derived from six *Streptomyces* strains, one *Micromonospora*, and one *Nocardia* strain. The matches for these eight sequences were to KSs responsible for the biosynthesis of three subgroups, respectively benzoisochromanequinones, angucyclines, and pentangular polyphenols. Specifically, one strain (S97) corresponded to benzoisochromanequinone subtype, three strains (S41, S71, and S107) were linked with angucycline subclass and four strains (S31, S40, S81, and S86) with spore pigment group. In addition, 10 sequences displayed < 93.6% similarity with their top matches, whose products could not be correlated with specific subtypes. Subsequent phylogenetic analysis also supported our clustering patterns based on maximum similarity.

**Table 3:** **KS_α_ amino acid sequences**.

**Strain**	**Nearest type strain**	**No. of unique clones**	**NCBI accession no**.	**Top BLAST match^a^ (source organism)**	**BLAST match pathway product^a^ (chemotype^b^)**	**Similarity (%)**
S12	*Kocuria gwangalliensis*	1	JX008015 AFO70129	ketosynthase (*Streptomyces cyaneus*)	Cur pigment (Pen)	85.2
S97*	*Micromonospora aurantiaca*	1	KJ094408 AHN91973	ketosynthase (*Streptomyces violaceoruber*)	Granaticin (Ben)	95.6
S107*	*Nocardia araoensis*	1	KJ094407 AHN91972	ketosynthase (*Streptomyces* sp.)	Unknown (Ang)	95.1
S78*	*Nocardiopsis alba*	2	JX008012 AFO70126	ketosynthase (*Streptomyces halstedii*)	sch pigment (Pen)	88.2
			JX008013 AFO70127	ketosynthase (*Streptomyces* sp.)	Benastatin (Pen)	89.2
S92*	*Nocardiopsis halotolerans*	1	KJ094409 AHN91974	ketosynthase (*Streptomyces tendae*)	Lysolipin (Pen)	88.7
S36*	*Saccharopolyspora gloriosa*	1	JX008003 AFO70117	ketosynthase (*Streptomyces antibioticus*)	Simocyclinone (Ang)	93.1
S33	*Salinispora arenicola*	1	JX008009 AFO70123	ketosynthase (*Streptomyces griseus*)	fredericamycin (Pen)	91.1
S34	*Salinispora tropica*	1	JX008010 AFO70124	ketosynthase (*Streptomyces griseus*)	fredericamycin (Pen)	90.1
S10*	*Streptomyces parvulus*	1	JX008008 AFO70122	ketosynthase (*Streptomyces antibioticus*)	simocyclinone (Ang)	92.1
S31	*Streptomyces carnosus*	1	JX008007 AFO70121	ketosynthase (*Streptomyces coelicolor*)	whiE pigment (Pen)	100
S39*	*Streptomyces djakartensis*	1	JX008002 AFO70116	ketosynthase (*Streptomyces* sp.)	sch 47554 (Ang)	93.1
S40	*Streptomyces luteosporeus*	1	JX008004 AFO70118	ketosynthase (*Streptomyces halstedii*)	sch pigment (Pen)	96.6
S41*	*Streptomyces rochei*	1	JX008005 AFO70119	ketosynthase (*Streptomyces ambofaciens*)	Unknown (Ang)	99.0
S71*	*Streptomyces anulatus*	1	JX008006 AFO70120	ketosynthase (*Streptomyces* sp.)	sch 47554 (Ang)	94.6
S72*	*Streptomyces xiamenensis*	1	JX008011 AFO70125	ketosynthase (*Actinomadura hibisca*)	Pradimicin (Ant)	88.2
S81	*Streptomyces diastaticus*	1	JX008014 AFO70128	ketosynthase (*Streptomyces coelicolor*)	whiE pigment (Pen)	98.0
S86	*Streptomyces griseorubens*	1	KJ094410 AHN91975	ketosynthase (*Streptomyces cyaneus*)	Cur pigment (Pen)	97.0

a *Top BLAST matches are to the KSα domains associated with experimentally characterized biosynthetic pathways of aromatic polyketides*.

b *Pen-Pentangular polyphenols, Ben-Benzoisochromanequinones, Ang-Angucyclines, Ant-Anthracyclines*.

The 10 strains marked with *were selected for small-scale fermentation.

### Small-scale fermentation and aromatic polyketide discovery

**Figure 3:**
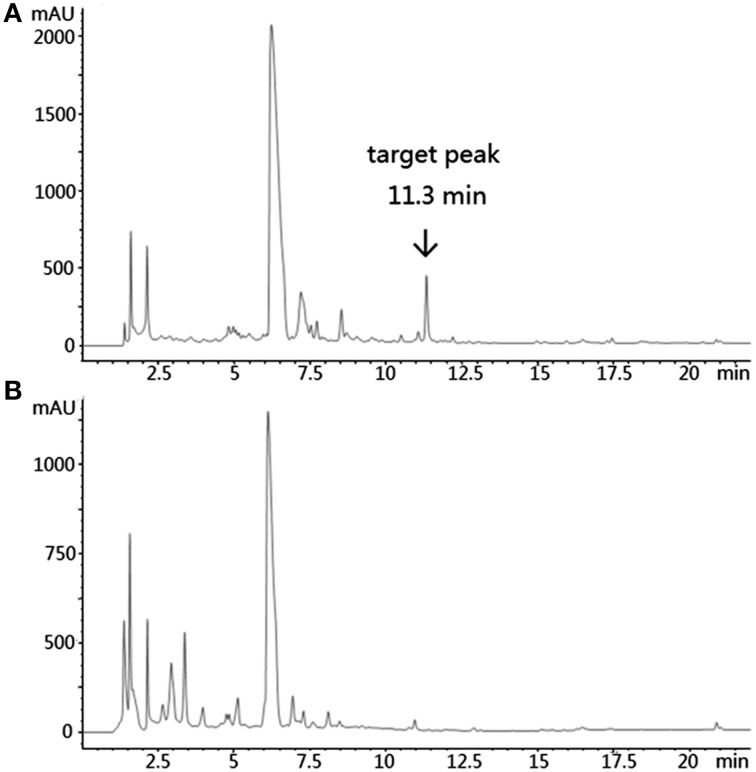
**HPLC of the ethyl acetate extract of ***S. anulatus*** S71 fermentation broth**. Target peak was eluted at 11.3 min **(A)**. HPLC of the ethyl acetate extract of broth medium as a negative control **(B)**. Detection wavelength: 210 nm.

**Figure 4:**
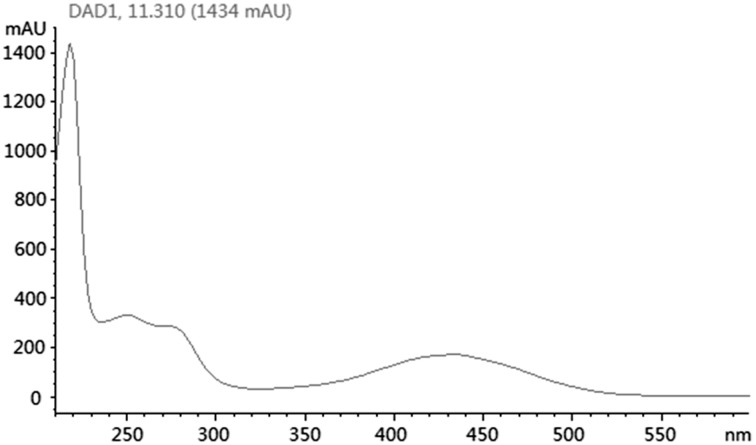
**UV/vis spectra of selected peak at t_R_ 11.3 min**.

**Figure 5:**
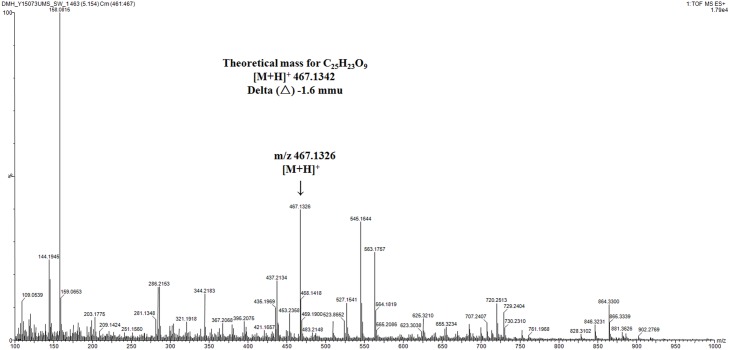
**High-resolution mass spectrum of selected ion at t_R_ 5.15 min in TIC**. mmu, milli-mass units.

**Figure 6:**
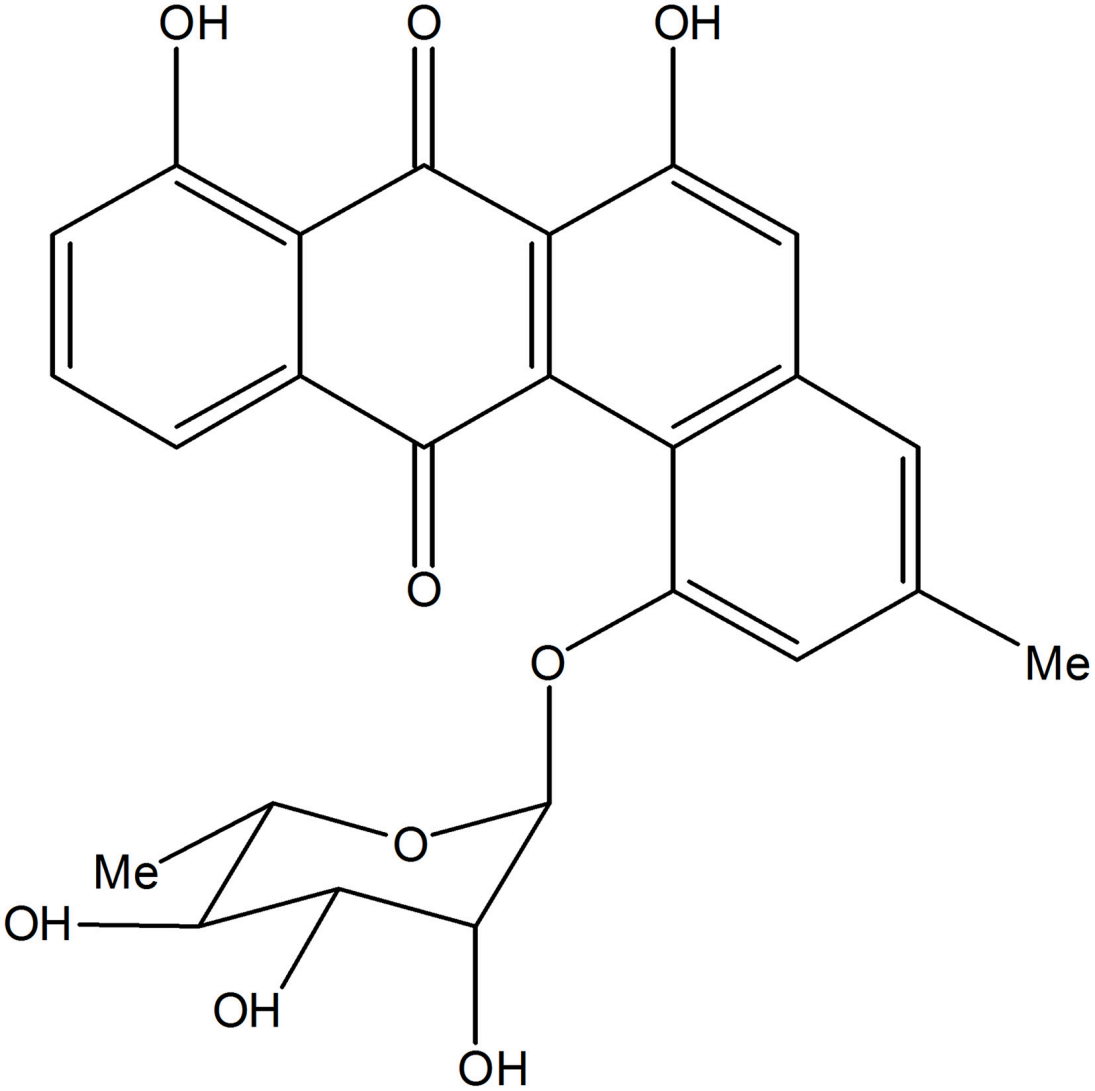
**Structure of amycomycin B**.

Based on KS_α_ sequence analysis, 10 strains were selected for small-scale fermentation (Table 3), among which one strain (*Micromonospora aurantiaca* S97) was used to test the production of putative benzoisochromanequinone, three strains (*Streptomyces rochei* S41, *Streptomyces anulatus* S71 and *Nocardia araoensis* S107) for putative angucyclines and other six strains (*Streptomyces parvulus* S10, *Saccharopolyspora gloriosa* S36, *Streptomyces djakartensis* S39, *Streptomyces xiamenensis* S72, *Nocardiopsis alba* S78, and *Nocardiopsis halotolerans* S92) for putative other subtypes. Expected products were preliminarily distinguished from the metabolite profiles according to their UV/vis absorption characteristics. Finally, one major metabolite present in the extract of *Streptomyces anulatus* strain S71 (Figure 3A) showed its UV-vis absorption (Figure 4) similar to that of typical angucyclines such as landomycin, which was absent in the control (Figure 3B). Subsequently, by using LC-MS, both HR ESI-MS ([M+H]^+^*m/z* = 467.1326) (Figure 5) and UV data (λ_max_: 252, 434 nm) (Figure 4) of the target substance almost corresponded to the data reported for one angucycline amycomycin B (HRESIMS: *m/z* 489.1154 [M+Na]^+^; UV λ_max_: 249, 427 nm) (Figure 6) (Guo et al., 2012), indicating that the detected compound was either amycomycin B itself or its analog. This finding indicated that *S. anulatus* S71 produced angucycline compound under the lab culture condition. Unfortunately, we did not detect any expected aromatic polyketide from other strains under lab fermentation condition.

## Discussion

In this study, comprehensive investigation of 15 sponge species and combination of five culture media led to the isolation of 20 actinobacterial genera. The isolation of indigenous marine genera (*Marihabitans, Salinispora*, and *Serinicoccus*) showed the marine characteristic of the actinomycetes from the South China Sea sponges. Actinobacteria are widely dispersed throughout the marine environments, including water column, marine organisms, marine snow, and sediments (Ward and Bora, 2006). Here, we respectively compare the culturable diversity of the South China Sea sponge-associated actinomycetes with that of marine sediment-derived, coral-associated, and seawater-derived actinomycetes (Table 4). It is apparent that the actinobacterial diversity in any individual habitat cannot cover the diversity revealed in present study. Specifically, among the 20 genera from the South China Sea sponges, one genus (*Marihabitans*) has not been found from marine sediments, four genera (*Marihabitans, Nonomuraea, Polymorphospora*, and *Streptomonospora*) not isolated from corals, and six genera (*Nonomuraea, Polymorphospora, Pseudonocardia, Saccharopolyspora, Salinispora*, and *Streptomonospora*) not cultured from seawater. Consequently, South China Sea sponges displayed their advantage as a prolific source of culturable actinomycetes compared with other marine habitats.

**Table 4:** **Comparison of the culturable diversity of South China Sea sponge-associated actinomycetes with that of marine sediment-derived, coral-associated, and seawater-derived actinomycetes**.

**South China Sea sponge-associated actinomycetes**	**Marine sediment-derived actinomycetes**	**Coral-associated actinomycetes**	**Seawater-derived actinomycetes**	**References**
*Arthrobacter*	+	+	+	Wietz et al., 2012; Yang et al., 2013; Zhang et al., 2014
*Brachybacterium*	+	+	+	Wang et al., 2010; Yang et al., 2013; Zhang et al., 2014
*Brevibacterium*	+	+	+	Wang et al., 2010; Yang et al., 2013; Zhang et al., 2014
*Kocuria*	+	+	+	Wang et al., 2010; Yang et al., 2013; Zhang et al., 2014
*Marihabitans*	−	−	+	Kageyama et al., 2008
*Microbacterium*	+	+	+	Wang et al., 2010; Yang et al., 2013; Zhang et al., 2014
*Micrococcus*	+	+	+	Harwati et al., 2007; Yang et al., 2013; Zhang et al., 2014
*Micromonospora*	+	+	+^*^	Chen et al., 2011; Yang et al., 2013
*Mycobacterium*	+	+	+	Al-Awadhi et al., 2012; Yang et al., 2013; Zhang et al., 2014
*Nocardia*	+	+	+^*^	Chen et al., 2011; Zhang et al., 2013b
*Nocardiopsis*	+	+	+^*^	Maldonado et al., 2005; Zhang et al., 2013b
*Nonomuraea*	+	−	−	Maldonado et al., 2005
*Polymorphospora*	+	−	−	Tamura et al., 2006
*Pseudonocardia*	+	+	−	Maldonado et al., 2005; Zhang et al., 2013b
*Rhodococcus*	+	+	+	Chen et al., 2011; Al-Awadhi et al., 2012; Yang et al., 2013
*Saccharopolyspora*	+	+	−	Maldonado et al., 2005; Zhang et al., 2013b
*Salinispora*	+	+^*^	−	Gontang et al., 2007
*Serinicoccus*	+	+^*^	+	Yi et al., 2004; Gontang et al., 2007
*Streptomonospora*	+	−	−	Zhang et al., 2013a
*Streptomyces*	+	+	+	Chen et al., 2011; Zhu et al., 2011; Yang et al., 2013

*+, The actinomycete genera are also cultivated from other marine habitats*.

*−, The actinomycete genera have not been cultivated from other marine habitats*.

* *16S rRNA gene sequences were submitted to GenBank but paper is unpublished*.

Prior to our study, 15 actinomycete genera have been cultivated from South China Sea sponges, including *Actinomadura, Catenuloplanes, Cellulosimicrobium, Gordonia, Micromonospora, Mycobacterium, Nocardia, Nocardiopsis, Pseudonocardia, Rhodococcus, Saccharomonospora, Salinispora, Sphaerisporangium, Streptomyces*, and *Verrucosispora*. By investigating as many as 15 previously unexplored South China Sea sponges, the known diversity of sponge-associated actinomycetes was significantly extended, with a total of 27 genera successfully cultivated (including previously reported 15 genera and newly cultivated 12 genera in this study). Excitingly, three rare genera (*Streptomonospora, Polymorphospora*, and *Marihabitans*) were isolated from marine sponges for the first time. *Streptomonospora* is a group of strictly halophilic filamentous actinomycetes in Nocardiopsaceae. *Streptomonospora* strains were previously derived from hypersaline soil (Cai et al., 2008) and salt lake (Cai et al., 2009). Until recently, two *Streptomonospora* strains were found from marine sediments, indicating its existence in the marine environment (Zhang et al., 2013a). *Polymorphospora* is a genus in *Micromonosporaceae*, and *Polymorphospora* strains were mainly isolated from soil surrounding mangrove roots (Tamura et al., 2006). *Marihabitans* is a genus in *Intrasporangiaceae* (Kageyama et al., 2008). Notably, the genus is quite rare and only one strain was previously cultured from surface seawater (Kageyama et al., 2008).

Over the past decade, actinomycetes have been intensively isolated from sponges inhabiting the Yellow Sea, the Caribbean Sea, the Red Sea, and the Mediterranean Sea as well (Abdelmohsen et al., 2014a). By comparing the diversity of the sponge-associated actinomycetes from the separate geographical locations, we found that different region generally harbored distinct sponge-associated actinomycetes, including both common actinomycete genera (*Micromonospora, Nocardiopsis, Rhodococcus*, and *Streptomyces*) and respective different actinomycete groups (Table 5). Notably, seven genera (*Catenuloplanes, Marihabitans, Polymorphospora, Saccharopolyspora, Serinicoccus, Sphaerisporangium*, and *Streptomonospora*) not found from the sponges in other oceans were cultivated from the South China Sea sponges, indicating the biogeographic variability in the South China Sea sponge-associated actinobacterial communities.

**Table 5:** **Comparison of the culturable diversity of the sponge-associated actinomycetes from the South China Sea, Yellow Sea, Caribbean Sea, Red Sea, and Mediterranean Sea**.

**Actinomycete genera**	**South China Sea sponge-associated actinomycetes**	**Yellow Sea sponge-associated actinomycetes**	**Caribbean sponge-associated actinomycetes**	**Red Sea sponge-associated actinomycetes**	**Mediterranean sponge-associated actinomycetes**
*Actinoalloteichus*	−	+	−	−	−
*Actinokineospora*	−	−	−	+	−
*Actinomadura*	+	+	−	−	−
*Arthrobacter*	+	−	−	+	−
*Blastococcus*	−	+	−	−	−
*Brachybacterium*	+	−	−	+	−
*Brevibacterium*	+	−	−	+	−
Catenuloplanes^*^	+	−	−	−	−
*Cellulosimicrobium*	+	−	+	−	−
*Corynebacterium*	−	−	−	+	+
*Curtobacterium*	−	−	−	+	−
*Dietzia*	−	−	−	+	−
*Georgenia*	−	+	−	−	−
*Gordonia*	+	+	−	−	+
*Kocuria*	+	−	−	+	+
Marihabitans^*^	+	−	−	−	−
*Microbacterium*	+	−	+	+	−
*Micrococcus*	+	−	−	+	−
*Micromonospora*	+	+	+	+	+
*Mycobacterium*	+	−	−	+	+
*Nocardia*	+	+	−	+	−
*Nocardiopsis*	+	+	−	+	+
*Nonomuraea*	+	+	−	−	−
Polymorphospora^*^	+	−	−	−	−
*Pseudonocardia*	+	+	−	−	−
*Rhodococcus*	+	+	−	+	+
*Rothia*	−	−	−	+	+
*Rubrobacter*	−	−	−	−	+
*Saccharomonospora*	+	−	−	+	−
Saccharopolyspora^*^	+	−	−	−	−
*Salinispora*	+	−	+	+	−
Serinicoccus^*^	+	−	−	−	−
*Solwaraspora*	−	−	+	−	−
Sphaerisporangium^*^	+	−	−	−	−
Streptomonospora^*^	+	−	−	−	−
*Streptomyces*	+	+	+	−	+
*Verrucosispora*	+	−	+	−	−

The genera marked with

* *were currently limited to South China Sea. The shading on rows highlight the sponge-associated actinomycete genera widely distributed in distinct oceans*.

The use of molecular approaches for describing microbial diversity has greatly enhanced the knowledge of population structure in sponge-associated bacterial communities. Diverse actinobacterial groups belonging to Actinobacteridae have been detected from various sponges (Simister et al., 2012). To our knowledge, at least 22 sponge-associated actinomycete genera have been revealed by molecular techniques, including *Actinomyces, Agromyces, Amycolatopsis, Arthrobacter, Brevibacterium, Cellulosimicrobium, Corynebacterium, Kocuria, Microbacterium, Micrococcus, Microlunatus, Micromonospora, Mycobacterium, Nocardioides, Nocardiopsis, Propionibacterium, Pseudonocardia, Rhodococcus, Ruania, Saccharopolyspora, Streptomyces*, and *Verrucosispora*. This number is much lower than that of the cultivated genera (60 genera) (Abdelmohsen et al., 2014a). Two factors are thought to lead to this result. First, the majority of the amplicon libraries were constructed using bacterial universal primers, thus it is difficult to detect those low-abundance actinobacterial groups. Second, environmental surveys based on 16S rRNA gene sequencing preferred to describe the bacterial community structure at the phylum level but not genus level. Therefore, the diversity of sponge-associated actinomycetes was mainly revealed by culture-based methods. Notably, to date several genera (*Actinomyces, Amycolatopsis, Microlunatus, Propionibacterium, Ruania*) detected by molecular techniques have not been isolated from sponges, suggesting that the diversity is still worth exploring in future.

Sponges contain diverse actinobacterial groups, however, the ecological functions of the actinobacteria are hardly known. Sponge-associated actinomycetes produce bioactive small molecules like their terrestrial counterparts do. The possibility cannot be excluded that some compounds play an important role in the chemical ecology of sponge hosts. Considering actinomycete-derived secondary metabolites commonly occur in a very low concentration, the compounds are difficult to be extracted directly from sponges. Consequently, exploring the metabolic potential of the sponge-associated actinomycete strains facilitates the discovery of novel bioactive molecules. Aromatic polyketides are known to be produced by a few taxa among diverse actinomycetes. Thus, knowing their taxonomic distribution facilitates the prioritization of strains for aromatic polyketide search and discovery. In this work, seven genera (*Kocuria, Micromonospora, Nocardia, Nocardiopsis, Saccharopolyspora, Salinispora*, and *Streptomyces*) were screened out as potential producers of aromatic polyketides, including both recognized and previously not recognized producers. Notably, strains related to *Streptomyces, Micromonospora, Nocardia, Nocardiopsis, Saccharopolyspora*, and *Salinispora* were known producers of aromatic polyketides (Sun et al., 2007; Perez et al., 2009; Ding et al., 2012; Sousa et al., 2012; Xie et al., 2012; Jensen et al., 2015). However, one genus (*Kocuria*) not traditionally associated with aromatic polyketide production was detected as well, suggesting that poorly studied genera may be potential producers of aromatic polyketides. To date, aromatic polyketides have not been isolated from strains related to *Kocuria*, therefore, their potential in aromatic polyketide biosynthesis deserves further exploration.

In recent years, phylogenetic prediction has been successfully applied in the discovery of type I polyketides (Gontang et al., 2010). By bioinformatic analyses of KS sequence the prediction was preliminarily made, and test for the production of target compounds was subsequently preformed to confirm the sequence-based analyses. Considering diverse tailoring enzymes involved in the aromatic polyketide biosynthesis (Schneider, 2005), we think it is not feasible to accurately predict target substance merely based on KS_α_ sequence analysis. However, due to the conserved property of KS_α_ domain, it is possible to correlate one KS_α_ sequence (one strain) with one specific subtype (Metsä-Ketelä et al., 2002). Among 17 representative strains, eight were specifically related to three subgroups, respectively angucyclines, benzoisochromanequinones, and spore pigments (Figure 2). The angucycline group is the largest group of aromatic polyketides, rich in chemical scaffolds and biological activities (Kharel et al., 2012). The benzoisochromanequinone group comprises fewer compounds than angucyclines but its members show a wide range of biological activities as well (Brimble et al., 1999). Additionally, other nine strains cannot be correlated with specific chemotypes (Figure 2). However, these strains should not be neglected because they potentially have the capacity to produce novel subtypes.

For the rapid identification of aromatic polyketides from crude culture extracts, it is critical to develop an efficient approach. At present, it is feasible to determine the elemental composition of compounds in mixtures and identify natural products using LC/MS and UV/vis spectra (Nielsen et al., 2011; El-Elimat et al., 2013). In the case of aromatic polyketides, UV/vis spectra provided important clues on the presence of unsaturated cyclohexanedione structure and polyphenolic ring system and thus indicated the compound type, and LC/MS analysis gave precise molecular weight and suggested molecular formula of target signal. Subsequently, the molecular formulas were used as queries to match those reported aromatic polyketides in database. If some compounds were retrieved, then their UV-vis absorption maxima are compared with target substance. Only when both UV/vis spectra and high-resolution molecular weight were consistent, the compound was identified as known one or its analog. This method avoided large-scale fermentation and purification processes, thus saved time and resource. It can be used as a dereplication protocol for aromatic polyketides and enhance the efficiency of discovering novel aromatic polyketides.

To our knowledge, actinomycete strains generally contain a number of biosynthetic gene clusters. However, only a few corresponding metabolites have been obtained until now. Apparently, the majority of the biosynthetic gene clusters are unexpressed under standardized laboratory conditions, which leads to a low efficiency in the discovery of their secondary metabolites. Similarly, it is also present in the aromatic polyketide discovery from the South China Sea sponge-associated actinomycetes. Surveying recent advances in microbial natural product discovery, we think two strategies can be considered to exclusively explore the metabolic potential of the strains. One is to try activating silent biosynthetic pathways through external cues, co-cultivation and stress since it has achieved great success in the natural product discovery from fungi and actinomycetes (Scherlach and Hertweck, 2009). The other is to apply genetic manipulation techniques such as gene cluster cloning and heterologous expression because it has shown unique advantage in harvesting rare skeletons of aromatic polyketides (Feng et al., 2011). They should be preferentially attempted in future work.

In summary, a total of 20 actinomycete genera were isolated from the South China Sea sponges, including three rare genera (*Marihabitans, Polymorphospora*, and *Streptomonospora*) found from sponges first time. Potential aromatic polyketide producers were distributed in seven genera (*Kocuria, Micromonospora, Nocardia, Nocardiopsis, Saccharopolyspora, Salinispora*, and *Streptomyces*). By small-scale fermentation, one angucycline compound was detected from one *Streptomyces* isolate. This work advanced our knowledge of sponge-associated actinomycetes regarding their diversity and biogeography, and revealed their potential in aromatic polyketide production.

## Author contributions

ZL and WS designed the study. LH identified the sponge samples. WS performed the experiments. WS and FZ analyzed the data. WS, ZL, and KL wrote the manuscript. All authors read and approved the final manuscript.

### Conflict of interest statement

The authors declare that the research was conducted in the absence of any commercial or financial relationships that could be construed as a potential conflict of interest.
