# Supplementary material for: Actinomycetes from the South China Sea sponges: isolation, diversity, and potential for aromatic polyketides discovery
Source: Front Microbiol. 2015 Oct 1;6:1048. doi: 10.3389/fmicb.2015.01048 (PMC4589764; doi:10.3389/fmicb.2015.01048)
Supplement: Supplementary file 1 [file Table1.DOCX]

**Table S1｜Composition of 5 media for the isolation of actinomycetes.**

| **Medium formula** | **References** |
| --- | --- |
| M1 10 g soluble starch, 4 g yeast extract, 2 g peptone, 18 g agar, and 1 l of artificial seawater  M2 2 ml glycerol, 8 g yeast extract, 4 g malt extract, 10 g manitol, 10 g glucose, 0.2mg ZnSO_4_, 0.2mg MnSO_4_, 0.2mg CuSO_4_, 2mg FeSO_4_, 18 g agar, and 1 l of artificial seawater  M3 0.1 g L-asparagine, 0.5 g K_2_HPO_4_, 0.001 g FeSO_4_, 0.1 g MgSO_4_, 2 g peptone, 4 g sodium propionate, 18 g agar, and 1 l of artificial seawater  M4 0.5 g yeast extract, 0.25 g tryptone, 0.75 g peptone, 0.5 g glucose, 0.5 g soluble starch, 0.3 g K_2_HPO_4_, 0.024 g MgSO_4_, 0.3 g sodium propionate, 18 g agar, and 1 l of artificial seawater  M5 6 ml glycerol, 1 g L-arginine, 1 g K_2_HPO_4_, 0.5 g MgSO_4_, 18 g agar, and 1 l of artificial seawater | Mincer et al. (2002)  This study  Zhang et al. (2006)  Abdelmohsen et al. (2010)  Mincer et al. (2002) |
